# Dynamics of Bacterial Community Composition in the Malaria Mosquito's Epithelia

**DOI:** 10.3389/fmicb.2015.01500

**Published:** 2016-01-05

**Authors:** Majoline T. Tchioffo, Anne Boissière, Luc Abate, Sandrine E. Nsango, Albert N. Bayibéki, Parfait H. Awono-Ambéné, Richard Christen, Geoffrey Gimonneau, Isabelle Morlais

**Affiliations:** ^1^UMR Maladies Infectieuses Et Vecteurs Écologie, Génétique, Évolution Et Contrôle, IRD 224- Centre National de la Recherche Scientifique 5290- UM1- UM2Montpellier, France; ^2^Laboratoire d'entomologie médicale, OCEAC-IRDYaoundé, Cameroon; ^3^Faculté de Médecine et des Sciences Pharmaceutiques, Université de DoualaDouala, Cameroon; ^4^Faculté des Sciences, Centre National de la Recherche Scientifique UMR 7138Nice, France; ^5^Laboratoire de Biologie Virtuelle, Faculté des Sciences, UMR 713, Université de NiceNice, France

**Keywords:** bacterial communities, malaria vector, mosquito tissues, pyrosequencing, dynamics

## Abstract

The *Anopheles* midgut hosts diverse bacterial communities and represents a complex ecosystem. Several evidences indicate that mosquito midgut microbiota interferes with malaria parasite transmission. However, the bacterial composition of salivary glands and ovaries, two other biologically important tissues, has not been described so far. In this study, we investigated the dynamics of the bacterial communities in the mosquito tissues from emerging mosquitoes until 8 days after a blood meal containing *Plasmodium falciparum* gametocytes and described the temporal colonization of the mosquito epithelia. Bacterial communities were identified in the midgut, ovaries, and salivary glands of individual mosquitoes using pyrosequencing of the 16S rRNA gene. We found that the mosquito epithelia share a core microbiota, but some bacteria taxa were more associated with one or another tissue at a particular time point. The bacterial composition in the tissues of emerging mosquitoes varied according to the breeding site, indicating that some bacteria are acquired from the environment. Our results revealed temporal variations in the bacterial community structure, possibly as a result of the mosquito physiological changes. The abundance of *Serratia* significantly correlated with *P. falciparum* infection both in the midgut and salivary glands of malaria challenged mosquitoes, which suggests that interactions occur between microbes and parasites. These bacteria may represent promising targets for vector control strategies. Overall, this study points out the importance of characterizing bacterial communities in malaria mosquito vectors.

## Introduction

Despite the role of commensal and mutualistic microorganisms in the ecology and behavior of medically important arthropods, such as mosquitoes, or tsetse flies, the nature of host-microbe interactions is still poorly understood (Buchner, 1965; Toh et al., 2006; Moran et al., 2008; Geiger et al., 2011; Zouache et al., 2011; Boissière et al., 2012). So far, most studies that have investigated the bacterial communities of mosquitoes have focused on the midgut compartment (Lindh et al., 2005; Cirimotich et al., 2011; Dinparast Djadid et al., 2011; Wang et al., 2011; Boissière et al., 2012; Osei-Poku et al., 2012; Terenius et al., 2012; Tchioffo et al., 2013). However, insect salivary glands and ovaries are also known as key organs for virus or parasite replication, but the bacterial content of these tissues has not been fully assessed (Minard et al., 2013). Studies are needed to make a complete inventory of the microbial communities associated with *Anopheline* species, to characterize their composition and structure and to provide a comprehensive overview of their ecology.

Gut-inhabiting bacteria have been shown to interfere with parasite transmission in the mosquito (Pumpuni et al., 1996; Straif et al., 1998; Yoshida et al., 2001; Gonzalez-Ceron et al., 2003; Riehle et al., 2007; Cirimotich et al., 2011; Boissière et al., 2012; Bando et al., 2013; Tchioffo et al., 2013). Microbes that mosquitoes carry can also confer a fitness gain on their hosts, influencing nutrition, reproduction, heat tolerance, and resistance to pathogens (Buchner, 1965; Montllor et al., 2002; Scarborough et al., 2005; Favia et al., 2007; Hedges et al., 2008; Pais et al., 2008). Interestingly, midgut bacterial communities are dominated by widely distributed taxa that appear to colonize hosts opportunistically (Wang et al., 2011; Boissière et al., 2012; Coon et al., 2014). Microbes that are widespread in a large range of hosts are thought to fulfill a functional niche, they may be dependent on the host for their diet (Engel and Moran, 2013). A better knowledge of mosquito associated microbiota is necessary to understand the role of the bacterial communities in the mosquito physiology and how they could serve to manipulate the mosquito susceptibility to pathogens (Favia et al., 2007; Riehle et al., 2007).

In this study, we investigated the composition of microbiota in the different mosquito epithelia, guts, ovaries, and salivary glands, of adult *Anopheles* female mosquitoes to fill the gap on the bacterial content of ovaries and salivary glands, two biologically important tissues whose inhabiting microbes had not been described before. Mosquitoes were collected in different breeding sites, raised to adults and females fed on *Plasmodium falciparum*-containing blood. We then performed pyrosequencing of the 16S rRNA gene to reveal bacterial diversity in the three epithelia of adult mosquitoes dissected at different time points, from emergence until 8 days after the infectious blood meal. The aim of this study was to follow the dynamics of the bacterial communities in the different tissues across the adult mosquito lifespan and to describe the temporal changes in the colonization of the mosquito epithelia.

## Materials and methods

### Ethics statement

All procedures involving human subjects used in this study were approved by the Cameroonian national ethics committee (statement 046/CNE/SE/2012). Written informed consent was obtained from a legal parent for each volunteer.

### Sample collection and DNA extraction

*Anopheles* mosquitoes were sampled in aquatic habitats as previously described (Boissière et al., 2012; Gimonneau et al., 2014). Immature stages were collected from four breeding sites in three localities: Nkolbisson (decimal geographical coordinates: 3.873703, 11.443654), Ahala (3.793829, 11.489730), and Nkolondom (3.953832, 11.494821) in peri-urban areas of Yaoundé (Cameroon). Mosquito larvae from Nkolbisson, Ahala, and Nkolondom 11 were sampled in temporary water collections sites, such as puddles and tire tracks. In Nkolondom 10, larval habitats were semi-permanent cultivation furrows created by the practice of agriculture. Larvae were collected with a dipper, transferred in a 5-L container and brought to the insectary at the Organisation de Coordination pour la lutte contre les Endémies en Afrique Centrale (OCEAC). Larvae were placed in plastic trays (25 × 25 × 8 cm) filled with the water from the breeding site without food addition. Anophelinae larvae were identified morphologically using taxonomic keys (Gillies and De Meillon, 1968) and the other specimens discarded. Pupae were collected during two consecutive days, placed into a sterile plastic cup containing 20 ml of water from the breeding site, and transferred to holding cages (30 × 30 cm). After emergence adult mosquitoes were maintained in standard insectary conditions (27 ± 2°C, 85 ± 5% RH, and 12 h light/dark) and provided with 6% sterile sucrose solution.

Adult female mosquitoes were fed on a single *P. falciparum* gametocyte carrier to avoid infection rate variability due to the blood donor. The gametocyte donor was identified during a parasitological survey carried out in primary schools of the area of Mfou in November 2013. The infectious feeding was performed as previously described (Boissière et al., 2012). Briefly, a 500 μl blood volume was drawn by venipuncture, centrifuged, and the serum replaced by an AB serum from a non-immune donor. The blood mixture was filled in a pre-warmed glass feeder and mosquitoes were allowed to feed through a Parafilm membrane for 35 min. Fully engorged females were kept in the insectary under standard conditions until dissections. Mosquitoes were dissected at 24 h and 8 days after the gametocyte-containing blood meal.

Prior dissecting, mosquitoes were surface sterilized in 70% ethanol for 5 min and rinsed twice in sterile PBS solution. Midguts, ovaries, salivary glands, and carcasses were kept separately and stored individually in 50 μl PBS at −20°C until processing. DNAs from mosquito's tissues were extracted using the DNeasy Blood & Tissue Kit from Qiagen (Valencia, CA) and quantified (Tecan's Infinite® 200 PRO NanoQuant, Seestrasse, Berlin). DNAs from mosquito carcasses were used to identify *An. coluzzii* and *An. gambiae* mosquitoes using the PCR-RFLP method as previously described (Gimonneau et al., 2014). Quantification of malaria infections in the midguts was performed using a *P. falciparum*-specific qPCR targeting a 120 bp fragment of the cox1 gene, as described in Boissiére et al. (2013). *Wolbachia* infections were checked using DNA extracts from female ovaries as previously described (Baldini et al., 2014). *Wolbachia* has been recently identified in *Anopheles* mosquitoes from Burkina Faso and we screened our population of *Anopheles* for natural *Wolbachia* infections using a *Wolbachia*-specific PCR assay to provide accurate data about its presence in our studied area.

### Pyrosequencing and data analysis

A total of 40 mosquito DNAs was submitted to 454 pyrosequencing. Among these, 28 had fed on the gametocyte-containing blood, nine mosquitoes were from dissections performed at 1 day post-blood-feeding (pbf) and 19 from 8 days pbf. Twelve additional mosquitoes dissected within the 24 h post-emergence were added for the analysis to obtain an accurate view of the microbiota dynamics, data for these 12 individuals were already published in our recent paper that compared the mosquito microbiota between *An. coluzzi* and *An. gambiae* (Gimonneau et al., 2014). For each mosquito, midgut, salivary glands and ovaries were processed separately, leading to a total of 120 samples.

A PCR amplicon library was generated for each individual DNA sample (midgut, ovaries or salivary glands) using the 343F-806R primer set (343F 5′- TACGGGAGGCAGCAG -3′ and 806R 5′- GGACTACCAGGGTATCTAAT -3′) targeting the V3-V4 hypervariable domain of the 16S ribosomal RNA gene. Amplified DNAs were processed as previously described (Gimonneau et al., 2014) and sequenced on a GS FLX Titanium platform (Roche, Basel, Switzerland) at Genoscreen (Lille, France). A total of 770,618 sequence reads (tags) were recovered and subjected to quality controls. All 454 sequences were deposited in Sequences Reads Archives (SRR1038490, SRR1038491, SRR1038492, and SRR1038493).

Tag extraction and filtering were conducted using our previously described pipeline (Gimonneau et al., 2014). Sequence reads were dereplicated and assigned according to Boissière et al. (2012), Gimonneau et al. (2014), at a k difference of 6. Reads were clustered into OTUs according to their consensus taxonomy. Rarefaction curves were obtained by using the function described by Jenna Jacobs (http://www.jennajacobs.org/R/rarefaction.html#work2010). The OTU analysis and diversity indices (richness, Simpson, and Shannon estimators) were computed under R statistical software version 2.15.2 (Team, 2013) using the Vegan (Oksanen et al., 2013) and BiodiversityR (Kindt and Coe, 2005) packages. Indices (Shannon, Simpson) and richness were calculated using values from the genus taxonomic rank, the lowest rank obtained with the 454 technology. The relative abundance of tag sequences assigned into clusters was visualized as a heatmap using STAMP (Parks et al., 2014).

### Statistical analysis

A principal component analysis (PCA) was performed using the R “ade4” and “ade4TkGUI” packages to identify hidden patterns of bacterial taxa distribution between the mosquito tissues (midgut, ovaries, and salivary glands) at the different stages [emerging, day 1 (D1) and day 8 (D8) post-blood-feeding] and among localities. Then, Welch's *t*-test was further used to compare diversity indexes between newly emerged mosquitoes and blood infected mosquitoes. Comparison of the bacterial communities between *P. falciparum* infected and non-infected mosquitoes was performed using the *t*-test with Welch's correction.

## Results

### Mosquitoes and tag recovery

Among the 40 field mosquitoes used in this study, 11 were from Ahala, 21 from Nkolondom, and 8 from Nkolbisson (Table S1).

The gametocytemia of the blood donor used for the mosquito feeding was of 85 gametocytes/μl and no trophozoites were seen after reading a Giemsa-stained blood smear against 1000 white blood cells. The qPCR assay on field mosquito midguts revealed that 57.1% (16/28) were infected. The number of *P. falciparum* genomes per infected mosquito was derived from standard curves as described earlier (Boissiére et al., 2013) and expressed as the number of genomes per mosquito. Mean number of parasites in infected midgut was 32,168 ± 25,156 genomes/mosquito.

The 454 pyrosequencing generated a total number of 770,618 sequences reads across the 120 samples (Table 1). After tag filtering and sorting, 547,091 sequences were obtained, representing 70% of the 454 reads. Among these tags, over 99% were successfully assigned.

**Table 1:** **Summary of pyrosequencing tags across the mosquito life stages and among tissues**.

	**Emerging mosquitoes**			**Twenty four hour post-blood-feeding**			**Eight days post-blood-feeding**		
	**Midgut**	**Ovaries**	**Sal. glands**	**Midgut**	**Ovaries**	**Sal. glands**	**Midgut**	**Ovaries**	**Sal. glands**
Sample number	12	12	12	9	9	9	19	19	19
Total reads	83,652	76,667	81,707	53,940	53,406	57,634	111,680	120,636	131,296
Dereplicated tags	55,457	51,458	51,047	34,991	36,533	39,121	84,788	96,381	100,746
Mean sequences	6971	6388	6808	5993	5934	6403	5877	6349	6566
Range	5677–9350	3124–13,333	2212–13,345	1285–10,594	2100–9148	3700–8517	3612–11,597	3377–20,559	2958–12,266
Assigned tags (%)	96.9	99.9	99.9	99.4	99.4	99.9	99.9	99.9	99.9
Phylum number	8	10	9	5	4	4	8	11	17
OTU (genus level)	154	176	132	70	92	80	144	184	204

The rarefaction curves reached a plateau at 3000 reads, indicating that the sampling effort was adequate to retrieve most OTUs (Figure S1). The bacterial flora of the mosquito tissues, midgut, ovaries and salivary glands, belonged to 16 different phyla, among which 4 contributed to more than 99% of the total microbiota: *Proteobacteria, Actinobacteria, Bacteroidetes*, and *Firmicutes*.

### Bacterial communities structure in the mosquito ovaries and salivary glands

**Figure 1:**
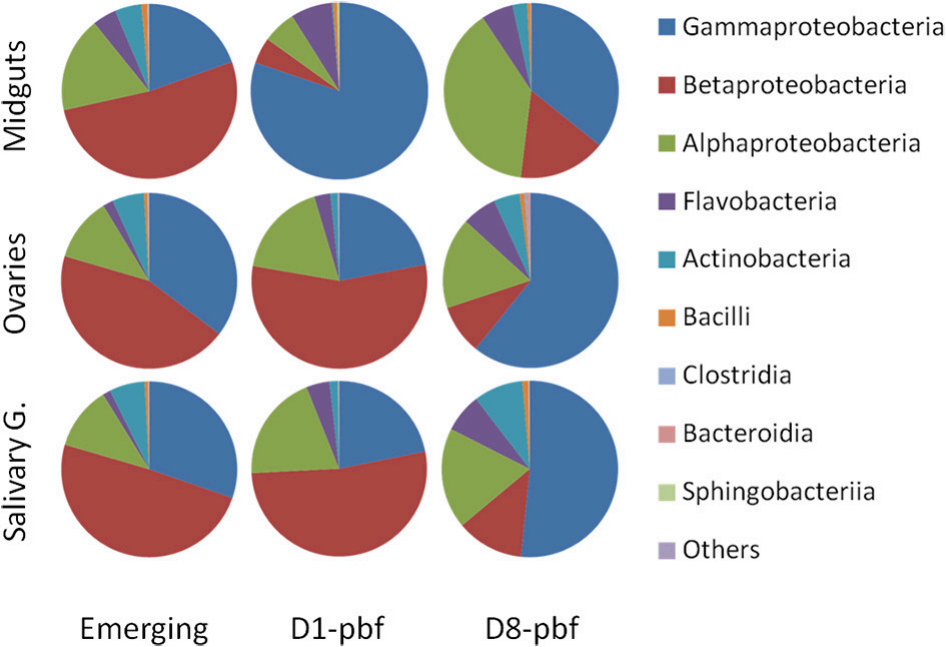
**Taxonomic classification of bacterial reads retrieved from the mosquito tissues at the different developmental stages**. Emerging, emerging mosquitoes; D1-pbf, 24 h post blood feeding; D8-pbf, 8 days post blood feeding. Taxonomic distributions are given at the Class level.

The bacterial communities in the mosquito ovaries and salivary glands yielded a similar succession pattern from emergence to day 8 post-blood-feeding (D8-pbf) when grouped on class-contribution (Figure 1). Comparison of the bacterial class abundance between ovaries and salivary glands using Welch *t*-test did not show significant differences at emergence and D1-pbf. At D8-pbf, the ovaries harbored significantly higher abundance of Gammaproteobacteria and lower abundance of *Actinobacteria* and *Betaproteobacteria* than salivary glands (Table S2). At this particular time point, D8-pbf, *Cedecea* were more abundant in the ovaries (7.4% by contrast to 0.3% in salivary glands) while *Comamonas, Brevibacterium*, and *Microbacterium* were enriched in salivary glands (Table S2, Figure S2).

The bacterial communities in ovaries and salivary glands of emerging mosquitoes are dominated by *Comamonas* (38.2 and 43.7%, respectively), *Acinetobacter* (18.0% and 14.5%, respectively), and *Pseudomonas* (13.5 and 12.7%, respectively) (Table S2). At D1-pbf, these genera remained major taxa but the other genera colonized the mosquito tissues: *Burkholderia* that represented < 5% of the bacterial content at emergence in the mosquito tissues increased up to 8.9 and 12.6% in ovaries and salivary glands, respectively; *Rhizobium* levels passed from 3.2 to 11.4% and 2.3 to 12.1% in ovaries and salivary glands, respectively; *Elizabethkingia* not identified in mosquitoes at emergence represented 2.9 and 3.9% of the sequences at 24 h after the blood-feeding. At D8-pbf, the mosquito tissues are mainly colonized by *Pseudomonas*, 41.5% in ovaries and 38.4% in salivary glands. The abundance of *Comamonas* dropped to 5.5 and 7.8% in ovaries and salivary glands, respectively, *Acinetobacter* represented about 5% of the sequences and *Burkholderia* < 2%. The bacterial densities of *Rhizobium* remained at the same level as at D1-pbf and *Elizabethkingia i*ncreased over 6%.

### Bacterial community structure in the mosquito midgut

**Figure 2:**
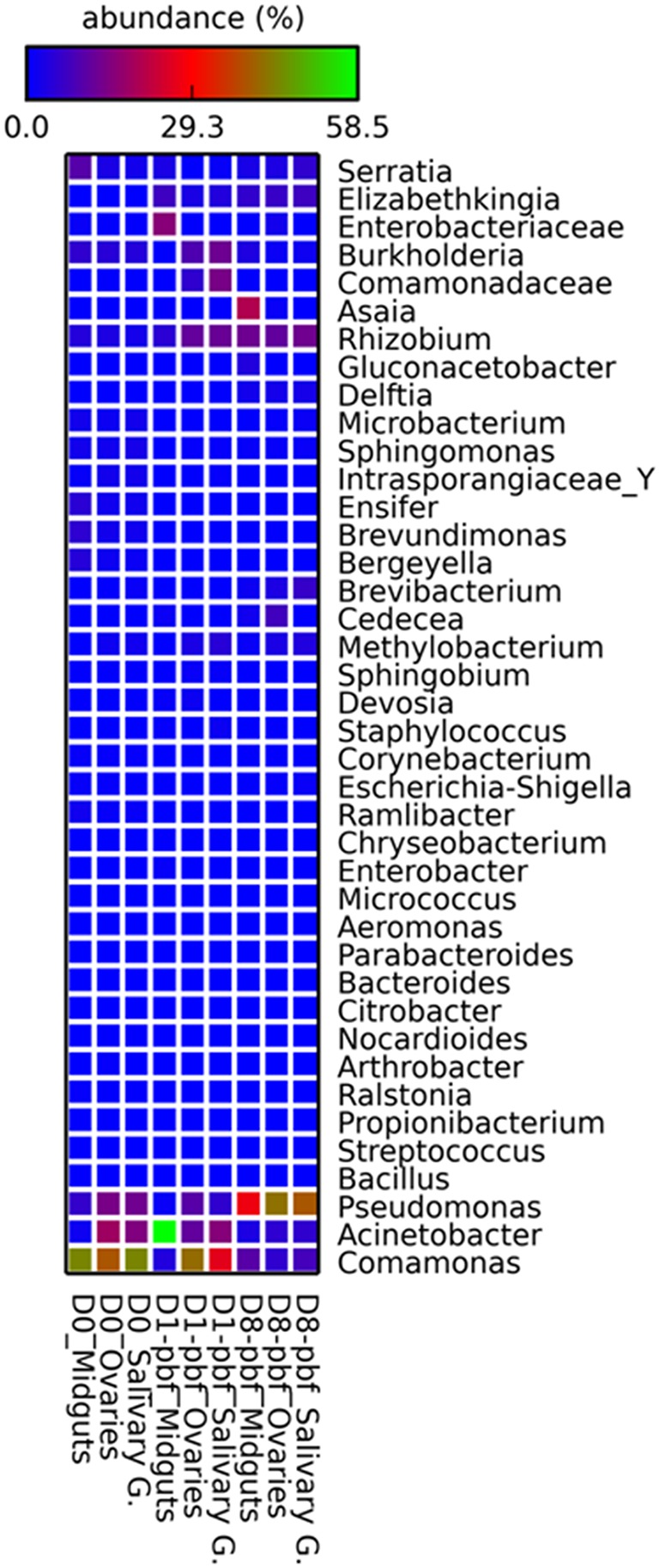
**Heatmap showing the relative abundance and distribution of the bacterial genera within the mosquito tissues for the different time points**. D0, emergence; D1-pbf, 24 h post blood feeding (pbf); D8-pbf, 8 days pbf; Salivary G, salivary glands.

The bacterial composition in the midgut appeared more distinct (Figures 1, 2 and Figure S2). If at emergence bacterial communities at class assignment were not different to the ones in the other tissues, differences appeared at the genera level. The gut bacterial content in emerging mosquitoes is dominated by *Comanomas* (43.6%), *Serratia* (9.8%), *Pseudomonas* (6.0%), *Burkolderia* (5.5%), and *Brevundimonas* (5.3%). At D1-pbf, *Gammaproteobacteria* had higher abundance, representing 80% of the total sequences in the guts, and *Acinetobacter, Enterobacteriaceae*, and *Elizabethkingia* represented the dominant taxa, 57.9, 15.5, and 7.2%, respectively. At D8-pbf, the abundance of *Gammaproteaobacteria* decreased and *Alphaproteobacteria* dominated, with 39% of the sequences of the gut (Figure 1). The bacterial communities at D8-pbf are mainly represented by *Pseudomonas* (27%), *Asaia* (20%), *Rhizobium* (12.4%), and *Comanomas* (10%).

The bacterial community structure encountered different shifts from one time-point to another. Some bacteria abundance decreased with time. *Comamonas* that accounted for 43.6% of the bacterial content at emergence only reached 4.1% of the tags at D1-pbf and 10.0% at D8-pbf. *Serratia*, representing 9.8% of tags at emergence, dropped to about 3% after the feeding. The abundance of other genera such as *Brevundimonas, Ensifer, Microbacterium, Arthrobacter, Ramlibacter, Bergeyella*, and the *Intrasporangiaceae* family, decreased to almost undetectable levels at D1-pbf and D8-pbf. For other bacteria, their relative abundance shifted only at D1-pbf. *Acinetobacter* that only accounted for 1.6% of the sequence at emergence and 3.3% at D8-pbf peaked at 57.9% of tag abundance at D1-pbf. *Enterobacteriaceae* that represented less that 1% of tag abundance at emergence and D8-pbf reached 15.5% 24 h after the blood-feeding. *Elizabethkingia* also peaked at this time-point, accounting for 7.2% of the tags. By contrast, some genera encountered a drop at D1-pbf, *Sphingomonas, Burkholderia, Corynebacterium, Microbacterium, Propionibacterium, Bacillus, Enterobacter*, and *Streptococcus* and returned to the emergence level at D8-pbf (Table S3). Finally, some bacteria genera were more abundant on older female midguts. *Gluconobacter* that were not identified in emerging and D1-pbf mosquitoes represented 3.9% of the sequences at D8-pbf. Other genera such as *Pseudomonas, Asaia, Rhizobium, Methylobacterium, Brevibacterium, Cedecea, Delftia* peaked at D8-pbf.

### Overall dynamics of the community structures and particularities

Richness and Shannon diversity indices indicated that the bacterial communities were significantly less diverse at D1-pbf for midguts and ovaries (Table 2), and the drop in the bacterial communities after the blood meal is particularly marked in the midgut, decreasing from 27 to 12 taxa per sample. Bacterial richness at emergence was higher in the midgut, however at D8-pbf, salivary glands harbored a more diverse microbiota than the gut. For all three tissues, bacteria genera such as *Sphingomonas, Brevundimonas, Ensifer, Ramlibacter, Bergeyella* reached almost undetectable levels after the blood-feeding. Other genera, *Bacillus, Corynebacterium, Microbacterium, Escherichia-Shigella*, had lower abundance only at D1-pbf and returned to emergence levels at D8-pbf. *Asaia, Delftia* mostly colonized the mosquito tissues of old females, *Gluconacetobacter* were only identified in tissues dissected at D8-pbf.

**Table 2:** **Bacterial diversity estimation indices in the mosquito tissues for each time point**.

**Time point**	**Midgut**			**Ovaries**			**Salivary glands**		
	**Richness**	**Shannon**	**Simpson**	**Richness**	**Shannon**	**Simpson**	**Richness**	**Shannon**	**Simpson**
Emerging	27	1.74	0.67	24	1.78	0.69	23	1.69	0.64
D1-pbf	12	1.03	0.47	16	1.57	0.71	18	1.76	0.77
D8-pbf	23	1.77	0.71	24	1.81	0.71	25	2.01	0.77

**Figure 3:**
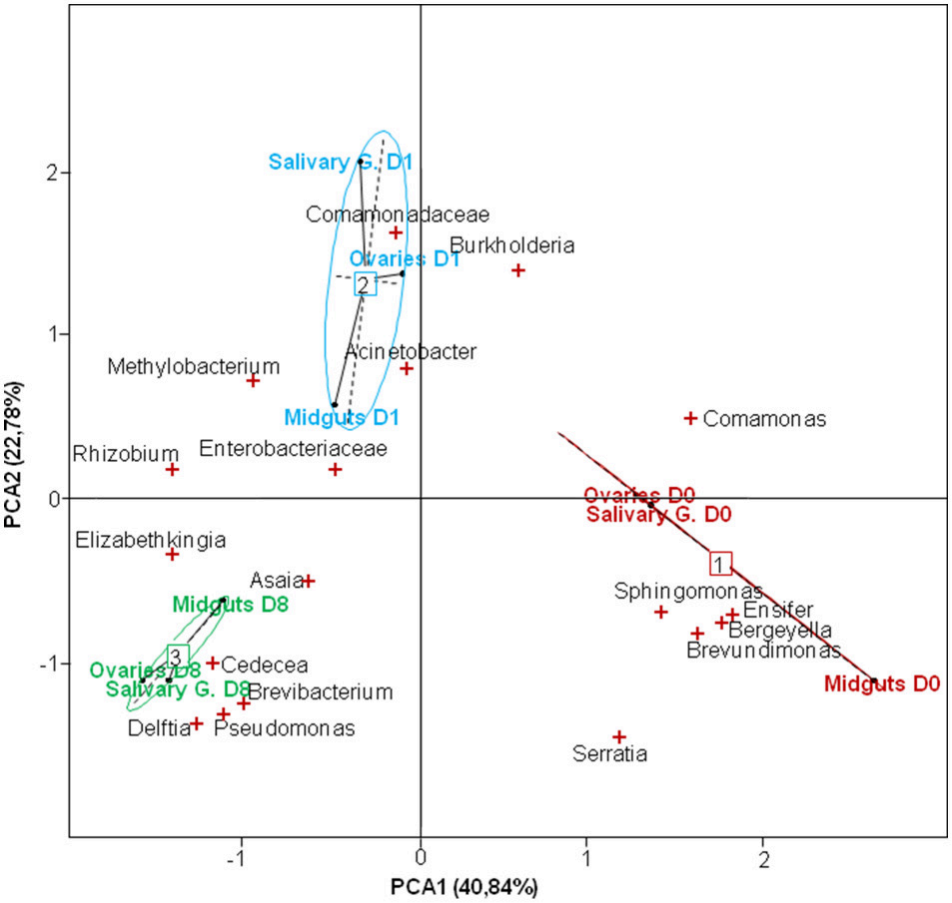
**Score-plot of the two first dimensions of the Principal Component Analysis (PCA) showing the distribution of bacterial genera in the mosquito tissues at different developmental stages**. D0, emergence; D1-pbf, 24 h post blood feeding (pbf); D8-pbf, 8 days pbf; Salivary G, salivary glands. The PCA revealed three clusters matching the developmental stages.

The PCA indicated close associations between tissues and bacteria at the different time points (Figure 3). Samples formed distinct clusters according to the time of collection, revealing significant changes in the microbial composition from one time point to another. *Asaia* were more abundant in the gut at D8-pbf while *Cedecea* were more represented at D8-pbf in ovaries, and members of the *Enterobacteriaceae* family in the mosquito midgut at D1-pbf. In addition, shifts in bacterial abundance after the bloodmeal were different from one tissue to another: the abundance of *Burkholderia* dropped in the midgut at D1-pbf while it increased in ovaries and salivary glands, *Elizabethkingia* colonized the gut earlier than the other tissues (Table S2, Figure 2). Minor bacteria genera were identified in a single mosquito tissue (25 in midguts, 21 in ovaries and 32 in salivary glands), they were recovered in only one sample at low abundance. As for the major bacteria, none were uniquely associated with a particular tissue.

**Figure 4:**
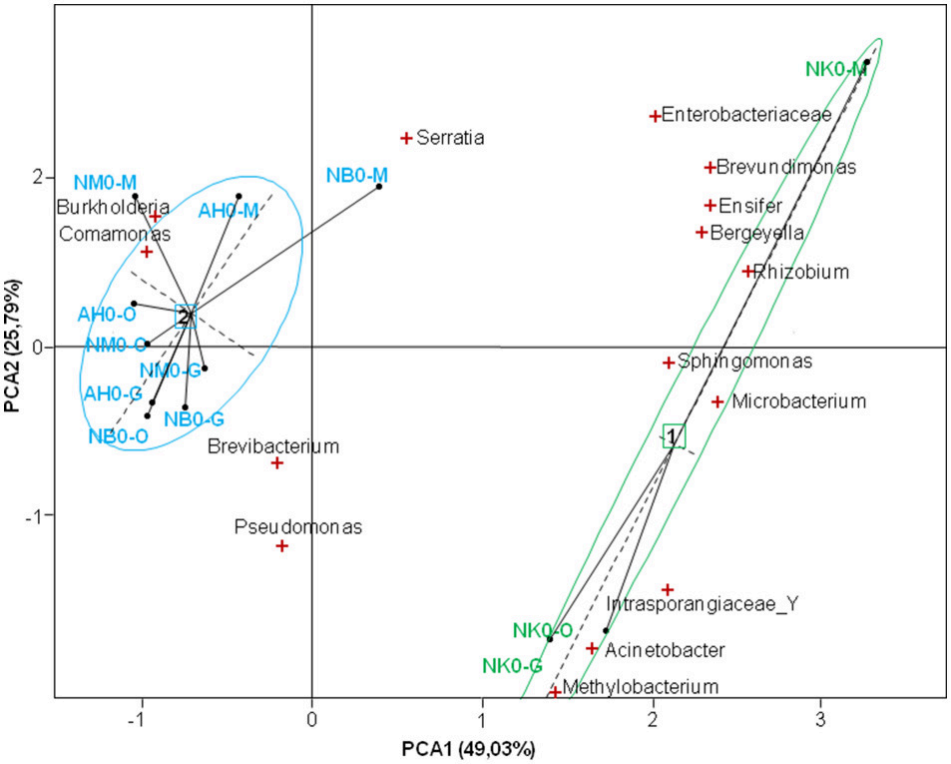
**Score-plot showing the distribution of bacterial genera in the tissues of mosquitoes for the different localities**. NK, Nkolondom 10; NM, Nkolondom 11; NB, Nkolbisson; AH, Ahala; M, midgut; O, ovaries; G, salivary glands.

We sequenced samples individually and great differences in taxa abundance were observed in mosquitoes according to the sampling site. Particularly, the bacterial content in the tissues of mosquitoes from Nkolondom 10 was distinct at emergence, enriched in *Acinetobacter* (Figure S2). The PCA yielded two main axes that accounted for 74.8% of the total variation in bacterial community structure among the different localities (Figure 4), and this indicates a considerable influence of the larval habitat type on the distribution of bacterial phylotypes. The single sequencing also highlighted individual variations in tag abundance or bacteria prevalence. *Brevundimonas* was found in all tissues of all mosquitoes at emergence, however the bacterium was not identified at D1-pbf and rather found at low level (<1%) at D8-pbf in 90% of the samples. The prevalence of *Asaia* increased over time and reached 94.7% in the gut samples at D8-pbf, the bacteria showed large variation in its relative abundance from one midgut sample to another, ranging from 0.05 to 68.9% of the sequenced tags.

We performed PCA to detect possible relationship between bacterial communities and *Anopheles* species but no correlation was highlighted (data not shown). Bacterial communities were not different between *An. gambiae* and *An. coluzzii*, as previously published (Gimonneau et al., 2014).

**Figure 5:**
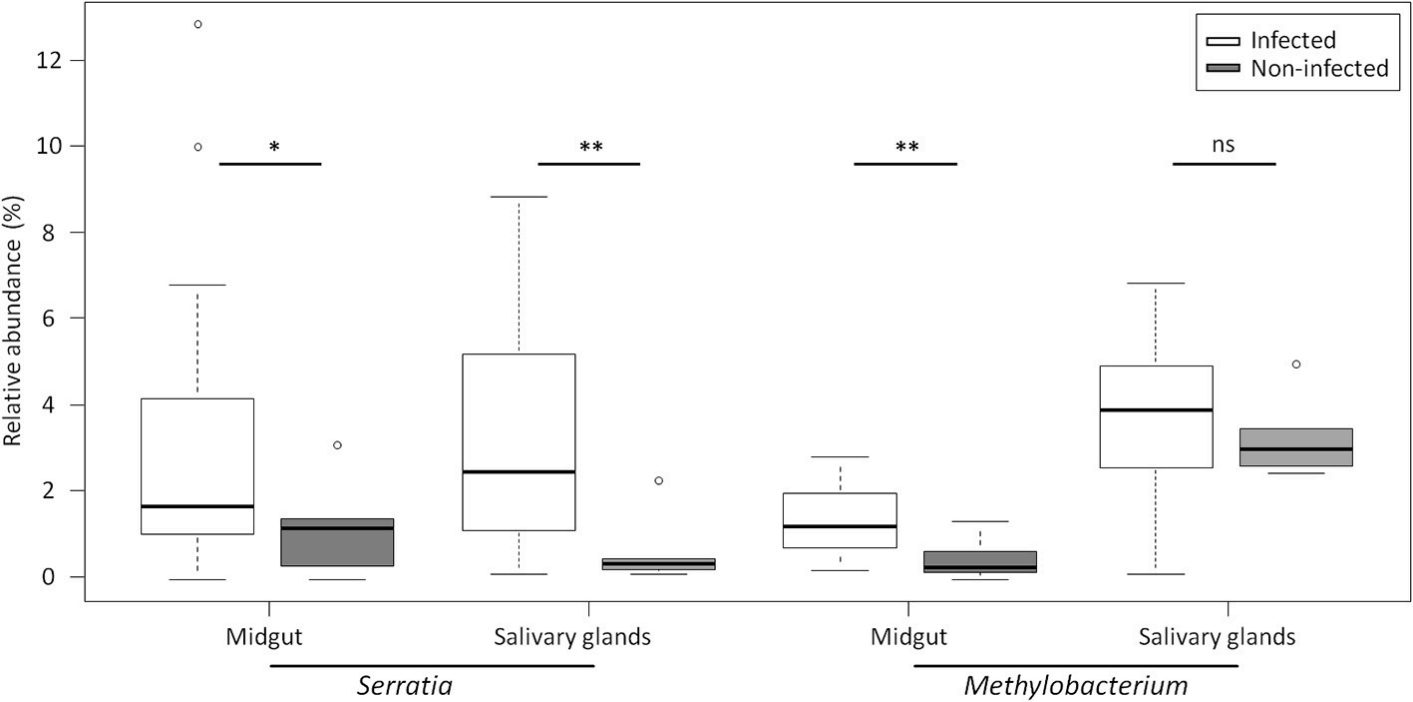
**Relative abundance of Serratia and Methylobacterium in ***P. falciparum*** infected and ***P. falciparum*** non- infected mosquitoes for both salivary glands and midgut**. The boxes represent the interquartile range (25–75th percentile), and the line within each box corresponds to the median value. ^*^P < 0.05, ^**^P < 0.01, and ns, non-significant. Circles represent outliers.

We investigated the presence of *Wolbachia* in our mosquitoes as *Wolbachia* infections were recently identified in single populations of *An. gambiae* s.s. in Burkina Faso (Baldini et al., 2014). No pyrotags were assigned to *Wolbachia* over all tissue samples. We then performed a *Wolbachia*-specific PCR on ovary templates to obtain a more sensitive detection of the bacteria, as *Wolbachia* infection increases with ovary maturation. We did not detect *Wolbachia* among the 40 ovary samples analyzed. We next compared the bacterial communities between *P. falciparum* infected and non-infected mosquitoes using the *t*-test with Welch's correction. No difference was observed in mosquito midgut at 24 h after the infected blood-meal. At D8-pbf, the relative abundance of *Methylobacterium* was significantly higher in *P. falciparum* positive midguts (1.37 ± 0.22 vs. 0.48 ± 0.29; *t* = 3.03, *P* < 0.01). Interestingly *Serratia* exhibited significant difference in abundances both in salivary glands and in midguts between infected and non-infected mosquitoes at D8-pbf (salivary glands: 3.46 ± 0.71 vs. 0.50 ± 0.33, *t* = 3.80, *P* < 0.01; midguts: 2.27 ± 0.66 vs. 0.82 ± 0.71, *t* = 2.14, *P* = 0.05; Figure 5).

## Discussion

We described here the microbiota diversity in salivary glands, ovaries and midguts of the *Anopheles* malaria mosquito. Microbial communities were recovered using pyrosequencing and we examined the microbial diversity in the different epithelia across the adult mosquito lifespan.

The adult mosquito midguts, ovaries and salivary glands were colonized by three dominant classes: *Gammaproteobacteria, Alphaproteobacteria*, and *Betaproteobacteria*. The relative abundance of the main bacterial genera varied among localities and mosquito samples but they were always among the major bacterial flora in the mosquito tissues. The core bacteria community was represented by *Pseudomonas, Comamonas, Acinetobacter, Rhizobium, Burkholderia*, and members of the *Enterobacteriaceae* family. Interestingly, we observed that the shifts in bacterial communities from emerging to aged (D8-pbf) mosquitoes were associated with abundance changes among already-present bacteria, and this indicates that the core microbiota persists over time.

The composition of the bacterial communities presented particular associations. Significant correlations were identified between the relative abundances of bacterial taxa and the mosquito nutritional status. Indeed, the PCA analyses clearly separated the microbiota into three different clusters according to the dissection time-point of mosquito samples. We have previously reported that the microbial composition of the different tissues of emerging mosquitoes is quite similar (Gimonneau et al., 2014). Here, we showed that the microbial communities in the epithelia are structured, possibly according to the physiological status of the mosquito. By contrast, and consistent with our previous study (Gimonneau et al., 2014), no distinct clustering was observed in the composition of the bacterial communities and the *Anopheles* species. *An. gambiae* and *An. coluzzii* shared similar microbiota.

Bacterial communities were not equally distributed in mosquito's epithelia, which may reflect tissue-bacteria adaptations. Bacteria can be acquired from the environment or transmitted from parents to offspring and their distribution within the mosquito tissues may be constrained by their mode of transmission (Kikuchi et al., 2007; Moran et al., 2008; Gimonneau et al., 2014). *Asaia* had a higher prevalence in the midgut, as compared to ovaries or salivary glands, and, by contrast, other bacteria such as *Methylobacterium, Comamonas, Acinetobacter*, were highly prevalent in the three epithelia. Interestingly, the structure of bacterial communities in ovaries and salivary glands was quite similar at the different time points. The bacterial assemblages in the two tissues may correspond to related temporal and physiological changes. In addition, we observed in samples from emerging mosquitoes a significant influence of the water collection site on bacteria distribution; the bacterial community structure differed greatly between mosquitoes sampled in temporary and semi-permanent breeding sites. This result confirms that the mosquito bacterial composition and diversity rely on environmental factors (Boissière et al., 2012; Gimonneau et al., 2014).

Changes in bacterial abundance after the blood meal were somehow different from one tissue to another, reflecting distinct evolution of the bacterial communities. The mosquito midgut undergoes the more drastic changes upon blood-feeding. *Acinetobacter, Elizabethkingia*, and members of the *Enterobacteriaceae* family represented the most abundant taxa (80%) 24 h after the blood meal. It is known that blood uptake induces dramatic changes in the midgut; the ingested blood carries large amounts of hemoglobin, catabolism of which results in a massive release of heme in the midgut, leading to oxidative stress conditions. In this study, the gut encountered the more severe drop in bacterial diversity and richness after blood feeding and certain bacteria such as *Ensifer, Arthrobacter*, or *Streptococcus* were not identified at this time point. Given that these bacteria were present at the other time points and that mosquitoes were only fed with sterile sugar after the blood meal, it is possible that the bacteria were at an undetectable level or alternatively, that PCR inhibitors such as hematin contained in the blood meal reduced the efficiency of their detection at D1-pbf. Conversely, in older mosquitoes, at D8-pbf, the salivary glands presented the highest bacterial diversity. Sharma et al. in 2014 reported a more diverse bacterial flora in salivary glands, as compared to the gut, in 3–4 day old sugar fed females of a laboratory colony of *An. culicifacies* (Sharma et al., 2014). However, in our study, when bacteria genera were found uniquely associated with a tissue, they were recovered in a single sample, at a very low abundance, which renders it difficult to determine whether these bacteria are associated with the given tissue or if they represent sampling bias.

We did not detect *Wolbachia* infections in the reproductive tissues of the adult females either with the high throughput sequencing of bacterial 16S rRNA gene or by using *Wolbachia*-specific qPCR. This could confirm that *Wolbachia* do not infect *Anopheles* mosquitoes in nature (Ricci et al., 2002). However, *Wolbachia* were recently identified at low frequency (10%) in field *An. gambiae* mosquitoes from Burkina Faso (Baldini et al., 2014). Interestingly, another recent study suggests that *Asaia* from the native mosquito microbiota impede *Wolbachia* transmission in *Anopheles* (Hughes et al., 2014). A larger sampling effort on a wider geographic area would be necessary to examine this putative correlation and determine whether the presence of *Asaia* in natural populations of *An. gambiae* interfere with the spread of *Wolbachia*. In our studied area, *Asaia* was frequently identified in the midgut (95%) as well as in the ovaries (45%) of aged mosquitoes and its high prevalence may reflect an important role in the mosquito biology.

We previously showed that *Enterobacteriaceae* loads were significantly higher in field-derived mosquitoes infected with *P. falciparum* and dissected at 8 days post-infection, suggesting a role of these bacteria in protecting the mosquito against malaria parasites in natural conditions (Boissière et al., 2012). In the present study, the abundance of *Serratia* was positively correlated to the mosquito infection status at D8-pbf both in the midgut and in salivary glands, two tissues involved in malaria parasite transmission. The correlation was not found at D1-pdf and this could be due to the early stage of infection, all ookinetes have not yet traversed the midgut epithelium, or to the small sample size. *Serratia* were identified in most tissue samples (95%) at low abundances, representing < 8% of the commensal microbiota. Different strains of *S. marcescens* were isolated from the midgut of wild mosquitoes from Burkina Faso and showed different ability to inhibit *P. berghei* development in co-infection experiments (Bando et al., 2013). Here, both *Methylobacterium* and *Serratia* abundances were higher in *P. falciparum*-infected mosquitoes, suggesting that malaria parasite transmission relies on the presence or absence of different key bacteria species.

## Conclusion

The current study described the dynamics of bacterial communities in the mosquito tissues, midguts, ovaries and salivary glands, at emergence, day 1, and day 8 upon blood feeding. To our knowledge, it is the first study providing an in-depth description of the microbiota diversity in salivary glands and ovaries of malaria mosquitoes. Our findings indicate that the mosquito epithelia share a core microbiota; however some bacteria taxa were more associated with one or another tissue at a particular time point. We revealed distinct patterns in the bacterial community structure between both the types of breeding site and the time of tissue collection. Furthermore, we identified a correlation between the abundance of *Serratia* and *P. falciparum* infection both in the midgut and salivary glands of malaria challenged mosquitoes. Our results point out the importance of characterizing mosquito bacterial communities in malaria endemic areas.

## Author contributions

IM and GG conceived and designed the experiments. MT, AB, ANB, SN, and GG performed the field experiments. PA provided facilities for experimental infections. MT, AB, and LA provided expertise in DNA extraction and laboratory facilities for pyrosequencing. MT and GG analyzed the data. RC provided expertise in Bioinformatics. MT, GG, and IM wrote the paper.

## Funding

This work was supported by funds from the Institut de Recherche pour le Développement (IRD), the Agence Nationale de la Recherche [ANR-11-BSV7-009-01 to IM], the APEGE programme from the Institut Ecologie et Environnement and the European Union Seventh Framework Programme (FP7) [GA 242095] and the ERC Starting Grant [GA260918].

### Conflict of interest statement

The authors declare that the research was conducted in the absence of any commercial or financial relationships that could be construed as a potential conflict of interest.

## Supplementary material

Table S1 **Origin and identification of mosquitoes. ID, mosquito identifier; Time-point, time of dissection**. In bold, *P. falciparum* positive mosquitoes.

Table S2 **Bacterial composition at the Class level for the different mosquito epithelia before and after blood feeding**.

Table S3 **Bacterial composition at the Genus level for the different mosquito epithelia before and after blood feeding**.

Figure S1 **Rarefaction analyses for each mosquito sample. (A)**, emerging mosquitoes; **(B)**, mosquitoes dissected at 24 h post blood feeding; **(C)**, mosquitoes dissected 8 days post blood feeding. Saturation curves were generated by plotting the number of unique sequence tags as a function of the number of randomly sampled tags. Tags were clustered at *k* = 6 differences. NK, Nkolondom 10; NM, Nkolondom 11; NB, Nkolbisson; AH, Ahala; 0, samples from emerging mosquitoes; 1, samples from mosquitoes dissected at 24 h post blood feeding; 8, samples from mosquitoes dissected 8 days post blood feeding; M, midgut; O, ovaries; G, salivary glands.

Figure S2 **Taxonomic classification of bacterial reads retrieved from each individual mosquito in the different tissues at the distinct developmental stages**. Emerging, emerging mosquitoes; D1-pbf, 24 h post blood feeding; D1-pbf, 8 days post blood feeding; NK, Nkolondom 10; NM, Nkolondom 11; NB, Nkolbisson; AH, Ahala. Relative abundances are given for the Genus level.
