# Supplementary material for: Dynamics of Bacterial Community Composition in the Malaria Mosquito's Epithelia
Source: Front Microbiol. 2016 Jan 5;6:1500. doi: 10.3389/fmicb.2015.01500 (PMC4700937; doi:10.3389/fmicb.2015.01500)
Supplement: Table S1 — Origin and identification of mosquitoes. ID, mosquito identifier; Time-point, time of dissection. In bold, P. falciparum positive mosquitoes. [file Table1.DOC]

**TABLE S1. Origin and identification of mosquitoes. ID, mosquito identifier ; Time-point, time of dissection. In bold, *P. falciparum* positive mosquitoes.**

| ID | Time-point | Collection site | Species |
| --- | --- | --- | --- |
| NK0-1 | emerging | Nkolondom 10 | *An. gambiae* |
| NK0-2 | emerging | Nkolondom 10 | *An. gambiae* |
| NK0-3 | emerging | Nkolondom 10 | *An. gambiae* |
| NM0-1 | emerging | Nkolondom 11 | *An. gambiae* |
| NM0-2 | emerging | Nkolondom 11 | *An. gambiae* |
| NM0-3 | emerging | Nkolondom 11 | *An. gambiae* |
| NB0-1 | emerging | Nkolbisson | *An. coluzzi* |
| NB0-2 | emerging | Nkolbisson | *An. coluzzi* |
| NB0-3 | emerging | Nkolbisson | *An. coluzzi* |
| AH0-1 | emerging | Ahala | *An. coluzzi* |
| AH0-2 | emerging | Ahala | *An. coluzzi* |
| AH0-3 | emerging | Ahala | *An. gambiae* |
| NK1-1 | D1-pbf | Nkolondom 10 | *An. gambiae* |
| NK1-2 | D1-pbf | Nkolondom 10 | *An. gambiae* |
| NK1-3 | D1-pbf | Nkolondom 10 | *An. gambiae* |
| NB1-1 | D1-pbf | Nkolbisson | *An. coluzzi* |
| NB1-2 | D1-pbf | Nkolbisson | ***An. coluzzi*** |
| NB1-3 | D1-pbf | Nkolbisson | *An. gambiae* |
| AH1-1 | D1-pbf | Ahala | ***An. coluzzi*** |
| AH1-2 | D1-pbf | Ahala | *An. coluzzi* |
| AH1-3 | D1-pbf | Ahala | ***An. coluzzi*** |
| NK8-1 | D8-pbf | Nkolondom 10 | ***An. gambiae*** |
| NK8-2 | D8-pbf | Nkolondom 10 | *An. gambiae* |
| NK8-3 | D8-pbf | Nkolondom 10 | *An. gambiae* |
| NK8-4 | D8-pbf | Nkolondom 10 | ***An. gambiae*** |
| NK8-5 | D8-pbf | Nkolondom 10 | *An. gambiae* |
| NK8-6 | D8-pbf | Nkolondom 10 | ***An. gambiae*** |
| NK8-7 | D8-pbf | Nkolondom 10 | ***An. gambiae*** |
| NK8-8 | D8-pbf | Nkolondom 10 | *An. gambiae* |
| NM8-1 | D8-pbf | Nkolondom 11 | *An. gambiae* |
| NM8-2 | D8-pbf | Nkolondom 11 | ***An. gambiae*** |
| NM8-3 | D8-pbf | Nkolondom 11 | ***An. gambiae*** |
| NM8-4 | D8-pbf | Nkolondom 11 | *An. gambiae* |
| NB8-1 | D8-pbf | Nkolbisson | ***An. gambiae*** |
| NB8-2 | D8-pbf | Nkolbisson | ***An. gambiae*** |
| AH8-1 | D8-pbf | Ahala | ***An. coluzzi*** |
| AH8-2 | D8-pbf | Ahala | ***An. coluzzi*** |
| AH8-3 | D8-pbf | Ahala | ***An. coluzzi*** |
| AH8-4 | D8-pbf | Ahala | ***An. coluzzi*** |
| AH8-5 | D8-pbf | Ahala | ***An. coluzzi*** |
