# Supplementary material for: Dynamics of Bacterial Community Composition in the Malaria Mosquito's Epithelia
Source: Front Microbiol. 2016 Jan 5;6:1500. doi: 10.3389/fmicb.2015.01500 (PMC4700937; doi:10.3389/fmicb.2015.01500)
Supplement: Table S2 — Bacterial composition at the Class level for the different mosquito epithelia before and after blood feeding. [file Table2.DOC]

**TABLE S2.** Bacterial composition at the Class level for the different mosquito epithelia before and after blood feeding.

| Class | **Midguts** | | |  | **Ovaries** | | |  | **Salivary glands** | | |
| --- | --- | --- | --- | --- | --- | --- | --- | --- | --- | --- | --- |
|  | **Emerging** | **D1-pbf** | **D8-pbf** |  | **Emerging** | **D1-pbf** | **D8-pbf** |  | **Emerging** | **D1-pbf** | **D8-pbf** |
| **Gammaproteobacteria** | **19.53**  **[17.28-21.78]** | **80.22**  **[77.18-83.26]** | **35.39**  **[32.41-38.38]** |  | **35.33**  **[32.17-38.49]** | **22.02**  **[21.04-22.99]** | **60.81**  **[59.04-62.57]** |  | **30.29**  **[26.50-34.08]** | **21.83**  **[19.67-23.99]** | **51.62**  **[50.24-53.00]** |
| **Betaproteobacteria** | **51.98**  **[46.84-57.12]** | **4.71**  **[3.99-5.44]** | **16.33**  **[12.71-19.95]** |  | **44.17**  **[39.47-48.87]** | **52.69**  **[52.07-59.31]** | **9.11**  **[8.40-9.82]** |  | **49.20**  **[44.03-54.38]** | **52.35**  **[48.27-56.42]** | **12.31**  **[11.31-13.31]** |
| **Alphaproteobacteria** | **17.69**  **[14.05-21.33]** | **6.11**  **[4.72-7.50]** | **38.96**  **[35.61-42.31]** |  | **11.78**  **[10.62-12.94]** | **17.66**  **[14.87-20.46]** | **16.83**  **[15.66-17.99]** |  | **11.69**  **[10.59-12.80]** | **19.76**  **[16.98-22.54]** | **18.52**  **[17.72-19.33]** |
| **Flavobacteria** | **4.39**  **[3.53-5.24]** | **7.53**  **[5.35-9.72]** | **5.87**  **[35.62-42.31]** |  | 1.88  [1.66-2.11] | **2.94**  **[2.85-3.03]** | **6.38**  **[5.89-6.86]** |  | 1.41  [1.29-1.53] | **4.26**  **[3.31-5.20]** | **7.09**  **[6.52-7.65]** |
| **Actinobacteria** | **4.89**  **[4.42-5.37]** | 0.22  [0.18-0.26] | **2.68**  **[2.36-3.00]** |  | **5.83**  **[5.18-6.48]** | 1.37  [1.22-1.51] | **4.80**  **[4.46-5.13]** |  | **6.51**  **[5.95-7.07]** | 1.58  [1.48-1.67] | **9.05**  **[8.64-9.46]** |
| **Bacilli** | **1.10**  **[0.75-1.45]** | 0.73  [0.43-1.04] | 0.62  [0.50-0.74] |  | 0.66  [0.54-0.79] | 0.03  [0.01-0.05] | 0.89  [0.71-1.07] |  | 0.65  [0.49-0.81] | 0.05  [0.04-0.07] | 1.08  [0.85-1.31] |
| Clostridia | 0.14  [0.11-0.17] | 0.01  [0.004-0.015] | 0.03  [0.02-0.04] |  | 0.10  [0.06-0.14] | 0.02  [0.01-0.03] | 0.38  [0.14-0.63] |  | 0.05  [0.03-0.07] | 0.01  [0.003-0.02] | 0.05  [0.03-0.08] |
| Bacteroidia | 0.03  [0.009-0.05] | 0 | 0.02  [0.001-0.035] |  | 0 | 0.005  [0-0.01] | 0.53  [0-1.34] |  | 0.01  [0-0.02] | 0.01  [0-0.03] | 0.01  [0.005-0.026] |
| Sphingobacteriia | 0.05  [0.03-0.06] | 0.41  [0.008-0.82] | 0.007  [0-0.019] |  | 0.05  [0.02-0.09] | 0.003  [0.000-0.006] | 0.01  [0.004-0.02] |  | 0.03  [0.02-0.04] | 0.01  [0.0002-0.02] | 0.10  [0.03-0.16] |
| unclass-Proteobacteria | 0.02  [0.007-0.029] | 0.01  [0.003-0.029] | 0 |  | 0.01  [0.004-0.02] | 0.23  [0.18-0.29] | 0.007  [0.00-0.01] |  | 0 | 0.08  [0.02-0.15] | 0.03  [0-0.09] |
| Epsilonproteobacteria | 0.005  [0-0.019] | 0 | 0 |  | 0.009  [0-0.02] | 0 | 0.1  [0-0.38] |  | 0.01  [0-0.04] | 0 | 0.002  [0-0.010] |
| Deltaproteobacteria | 0.03  [0.009-0.048] | 0 | 0.01  [0.003-0.032] |  | 0.04  [0.00-0.08] | 0 | 0.02  [0-0.03] |  | 0.03  [0-007-0.05] | 0 | 0.01  [0.002-0.03] |
| Fusobacteria | 0.05  [0-0.14] | 0 | 0.004  [0-0.015] |  | 0.005  [0-0.02] | 0 | 0.05  [0-0.13] |  | 0 | 0 | 0.01  [0-0.03] |
| Other | 0.05  [0.04-0.07] | 0 | 0.04  [0.007-0.069] |  | 0.07  [0.04-0.09] | 0 | 0.05  [0.02-0.07] |  | 0.05  [0.04-0.07] | 0.02  [0.003-0.04] | 0.04  [0.03-0.06] |
