# Supplementary material for: Dynamics of Bacterial Community Composition in the Malaria Mosquito's Epithelia
Source: Front Microbiol. 2016 Jan 5;6:1500. doi: 10.3389/fmicb.2015.01500 (PMC4700937; doi:10.3389/fmicb.2015.01500)
Supplement: Table S3 — Bacterial composition at the Genus level for the different mosquito epithelia before and after blood feeding. [file Table3.DOC]

**TABLE S3.** Bacterial composition at the Genus level for the different mosquito epithelia before and after blood feeding.

| *Genera* | **Midguts** | | |  | **Ovaries** | | |  | **Salivary glands** | | |
| --- | --- | --- | --- | --- | --- | --- | --- | --- | --- | --- | --- |
|  | **Emerging** | **D1-pbf** | **D8-pbf** |  | **Emerging** | **D1-pbf** | **D8-pbf** |  | **Emerging** | **D1-pbf** | **D8-pbf** |
| ***Pseudomonas*** | **6.02**  **[4.74-7.29]** | **0.90**  **[0.65-1.15]** | **26.98**  **[23.65-30.30]** |  | **13.52**  **[12.66-14.38** | **10.05**  **[6.14-13.96]** | **41.53**  **[38.56-44.51]** |  | **12.69**  **[11.64-13.74]** | **5.57**  **[3.99-7.16]** | **38.41**  **[35.84-40.98]** |
| ***Comamonas*** | **43.6**  **[38.46-48.74]** | **4.06**  **[3.39-4.72]** | **10.01**  **[6.69-13.32]** |  | **38.25**  **[33.84-42.67]** | **40.8**  **[37.09-44.51]** | **5.50**  **[4.977-6.02]** |  | **43.74**  **[38.72-48.77]** | **25.82**  **[24.00-27.64]** | **7.80**  **[7.08-8.59]** |
| ***Acinetobacter*** | **1.62**  **[1.25-1.97]** | **57.87**  **[53.11-62.63]** | **3.29**  **[2.63-3.95]** |  | **17.98**  **[14.33-21.62]** | **11.14**  **[9.06-13.22]** | **5.19**  **[3.80-6.58]** |  | **14.52**  **[10.77-18.28]** | **15.19**  **[12.65-17.74]** | **5.51**  **[3.86-7.16]** |
| ***Rhizobium*** | **4.14**  **[3.09-5.19]** | **4.79**  **[3.55-6.03]** | **12.44**  **[10.74-14.14]** |  | **3.22**  **[2.84-3.22]** | **11.38**  **[9.37-13.4]** | **11.18**  **[9.96-12.39]** |  | **2.28**  **[1.94-2.62]** | **12.11**  **[9.19-15.04]** | **12.74**  **[11.71-13.76]** |
| ***Burkholderia*** | **5.52**  **[4.84-6.19]** | 0.29  [0.20-0.37] | **3.50**  **[1.73-5.27]** |  | **4.54**  **[3.95-5.12]** | **8.88**  **[7.81-9.95]** | 1.16  [0.99-1.33] |  | **4.13**  **[3.62-4.64]** | **12.56**  **[10.52-14.61]** | 1.74  [1.39-2.09] |
| ***Elizabethkingia*** | 0.01  [0.007-0.016] | **7.16**  **[4.73-9.57]** | **5.80**  **[5.21-6.40]** |  | 0 | **2.93**  **[2.61-3.25]** | **6.34**  **[5.77-6.91]** |  | 0 | **3.92**  **[3.01-4.82]** | **9.16**  **[6.31-7.59]** |
| ***Cedecea*** | 0.08  [0.04-0.13] | 1.24  [0.41-2.06] | 2.00  [0.12-3.88] |  | 0.007  [0-0.013] | 0.08  [0-0.67] | **7.43**  **[2.25-12.62]** |  | 0.006  [0-0.01] | 0.06  [0.03-0.08] | 0.33  [0.11-0.55] |
| ***Methylobacterium*** | 0.14  [0.11-0.16] | 0.18  [0.12-0.24] | 1.01  [0.87-1.14] |  | 1.64  [1.35-1.92] | **3.04**  **[2.68-3.41]** | **3.05**  **[2.84-3.26]** |  | 1.83  [1.44-2.22] | **4.54**  **[3.80-5.28]** | **3.74**  **[3.48-3.99]** |
| ***Serratia*** | **9.78**  **[7.86-11.71]** | **3.00**  **[2.23-3.78]** | **2.79**  **[2.15-3.44]** |  | **2.93**  **[2.23-3.63]** | 0.32  [0.26-0.39] | **3.23**  **[2.50-3.95]** |  | **2.18**  **[1.75-2.61]** | 0.69  [0.50-0.88] | **5.55**  **[4.57-6.54]** |
| ***Brevibacterium*** | 0.15  [0.11-0.18] | 0.012  [0.007-0.017] | 1.40  [1.17-1.63] |  | 0.26  [0.23-0.30] | 0.08  [0-1.33] | **3.19**  **[2.95-3.43]** |  | 0.90  [0.73-1.07] | 0.08  [0.05-0.11] | **6.16**  **[5.71-6.61]** |
| ***Comamonadaceae*** | 0.17  [0.14-0.19] | 0.22  [0.11-0.32] | 0.27  [0.15-0.38] |  | 0.26  [0.2-0.31] | **5.49**  **[4.23-6.75]** | 0.07  [0.05-0.09] |  | 0.21  [0.17-0.24] | **13.58**  **[12.24-15.01]** | 0.09  [0.07-0.11] |
| ***Enterobacteriaceae*** | 0.96  [0.61-1.30] | **15.48**  **[9.36-21.6]** | 0.17  [0.11-0.23] |  | 0.32  [0.20-0.43] | 0.24  [0.11-0.37] | 2.28  [0.46-3.99] |  | 0.35  [0.24-0.46] | 0.15  [0.12-0.19] | 0.23  [0.15-0.30] |
| *Sphingomonas* | 2.17  [2.00-2.34] | 0.04  [0.02-0.07] | 0.68  [0.58-0.78] |  | 1.93  [1.78-2.07] | 0.57  [0.46-0.67] | 0.88  [0.80-0.97] |  | 2.66  [2.52-2.80] | 0.62  [0.48-0.75] | 1.00  [0.92-1.08] |
| *Delftia* | 0.05  [0.03-0.06] | 0.08  [0.04-0.12] | 2.06  [1.77-2.35] |  | 0.006  [0.001-0.011] | 0 | 2.31  [2.11-2.51] |  | 0.04  [0.02-0.06] | 0 | 2.60  [2.41-2.79] |
| *Brevundimonas* | 5.34  [3.97-6.71] | 0 | 0.19  [0.15-0.24] |  | 2.102  [1.88-2.32] | 0 | 0.31  [0.25-0.36] |  | 1.93  [1.73-2.13] | 0 | 0.31  [0.24-0.38] |
| ***Microbacterium*** | **2.21**  **[1.90-2.51]** | **0** | **0.44**  **[0.37-0.51]** |  | **1.525**  **[1.13-1.91]** | **0.12**  **[0.07-0.17]** | **0.54**  **[0.47-0.62]** |  | **2.10**  **[1.95-2.26]** | **0.09**  **[0.06-0.12]** | **1.18**  **[1.09-1.27]** |
| *Intrasporangiaceae* | 0.78  [0.60-0.96] | 0.10  [0.08-0.12] | 0.14  [0.11-0.17] |  | 2.12  [1.68-2.55] | 0.44  [0.39-0.50] | 0.17  [0.14-0.20] |  | 2.14  [1.72-2.57] | 0.57  [0.48-0.66] | 0.28  [0.24-0.32] |
| *Ensifer* | 4.84  [2.94-6.75] | 0 | 0.16  [0.03-0.29] |  | 1.84  [1.52-2.16] | 0.003  [0-0.007] | 0.01  [0.006-0.02] |  | 1.92  [1.61-2.23] | 0.01  [0.002-0.019] | 0.05  [0.03-0.07] |
| *Bergeyella* | 4.26  [2.82-5.71] | 0 | 0 |  | 1.74  [1.34-2.13] | 0.007  [0-0.02] | 0 |  | 1.30  [1.09-1.51] | 0.007  [0.0006-0.015] | 0.01  [0-0.025] |
| ***Asaia*** | 0.10  [0-0.20] | 0.28  [0.14-0.41] | **19.97**  **[13.19-26.75]** |  | 0.005  [0-0.01] | 0.02  [0-0.05] | 1.06  [0-2.32] |  | 0 | 0.01  [0.006-0.03] | 0.16  [0.004-0.315] |
| *Escherichia-Shigella* | 0.82  [0.62-1.02] | 0.45  [0.2-0.7] | 0.27  [0.18-0.35] |  | 0.46  [0.35-0.56] | 0.06  [0-0.24] | 0.59  [0.37-0.81] |  | 0.37  [0.29-0.45] | 0.06  [0.05-0.07] | 0.92  [0.59-1.24] |
| *Sphingobium* | 0.24  [0.18-0.29] | 0.09  [0.05-0.13] | 0.05  [0.03-0.07] |  | 0.36  [0.29-0.44 | 0.92  [0.59-1.25] | 0.06  [0.04-0.08] |  | 0.28  [0.22-0.34] | 0.69  [0.46-0.91] | 0.06  [0.04-0.07] |
| *Devosia* | 0.25  [0.20-0.29] | 0.73  [0.35-1.12] | 0.17  [0.11-0.23] |  | 0 | 1.45  [1.09-1.81] | 0.005  [-0-0.01] |  | 0.28  [0.20-0.36] | 0.0  [0.01-0.04] | 0.36  [0.20-0.51] |
| *Corynebacterium* | 0.79  [0.59-1.00] | 0.02  [0.01-0.03] | 0.23  [0.18-0.27] |  | 0.48  [0.39-0.56] | 0.02  [0.01-0.04 | 0.24  [0.17-0.31] |  | 0.54  [0.37-0.72] | 0.11  [0.08-0.12] | 0.29  [0.23-0.36] |
| *Arthrobacter* | 0.17  [0.02-0.32] | 0 | 0.009  [0-0.02] |  | 0.63  [0-1.47] | 0.08  [0.04-0.11] | 0.01  [0.004-0.03] |  | 0.01  [0.004-0.02] | 0.08  [0.003-0.174] | 0.03  [0-0.07] |
| *Ramlibacter* | 1.97  [0.83-3.10] | 0 | 0 |  | 0.74  [0.43-1.04] | 0 | 0 |  | 0.74  [0.36-1.11] | 0 | 0.003  [0-0.007] |
| *Bacillus* | 0.42  [0.07-0.77] | 0 | 0.25  [0.15-0.36] |  | 0.17  [0.09-0.25] | 0 | 0.27  [0.19-0.36] |  | 0.12  [0.06-0.18] | 0.005  [0.0009-0.01] | 0.26  [0.11-0.42] |
| *Streptococcus* | 0.26  [0.12-0.41] | 0 | 0.11  [0.04-0.18] |  | 0.14  [0.09-0.19] | 0.02  [0.01-0.04] | 0.24  [0.12-0.36] |  | 0.09  [0.05-0.14] | 0.007  [0.001-0.013] | 0.23  [0.12-0.34] |
| *Staphylococcus* | 0 | 0.64  [0.34-0.94] | 0.003  [-0-0.008] |  | 0.17  [0.12-0.22] | 0 | 0.24  [0.12-0.35] |  | 0 | 1.43  [0.81-2.04] | 0.004  [0-0.010] |
| *Propionibacterium* | 0.28  [0.23-0.33] | 0.004  [0.001-0.007] | 0.11  [0.07-0.14] |  | 0.28  [0.18-0.38] | 0.01  [0-0.05] | 0.13  [0.10-0.17] |  | 0.18  [0.12-0.24] | 0.06  [0.04-0.08] | 0.25  [0.18-0.33] |
| *Nocardioides* | 0.13  [0.11-0.16] | 0.01  [0.003-0.028] | 0.01  [0.004-0.02] |  | 0.283  [0.22-0.34] | 0.19  [0.14-0.24] | 0.01  [0.006-0.022] |  | 0.38  [0.33-0.43] | 0.08  [0.05-0.11] | 0.018  [0.008-0.029] |
| *Parabacteroides* | 0.004  [0-0.01] | 0 | 0.003  [0-0.01] |  | 0 | 0 | 0.25  [0-1.06] |  | 0 | 0.01  [0-0.04] | 0.006  [0-0.02] |
| *Ralstonia* | 0.58  [0.29-0.87] | 0.01  [0.004-0.016] | 0.01  [0.002-0.03] |  | 0.323  [0.24-0.39] | 0.1  [0.07-0.12] | 0.003  [0-0.007] |  | 0.26  [0.19-0.33] | 0.12  [0.09-0.15] | 0.01  [0.004-0.022] |
| *Citrobacter* | 0.03  [0.01-0.05] | 0.03  [0-0.07] | 0.01  [0-0.03] |  | 0 | 0 | 0.22  [0-0.49] |  | 0.02  [0.002-0.04] | 0.01  [0-0.021] | 0.03  [0.01-0.05] |
| *Gluconacetobacter* | 0 | 0 | 3.87  [1.3-6.45] |  | 0 | 0 | 0.01  [0-0.03] |  | 0 | 0 | 0.12  [0.01-0.23] |
| *Enterobacter* | 0.003  [0-0.008] | 0.76`  [0.21-1.31] | 0 |  | 0 | 0.01  [0.004-0.016] | 0.10  [0-0.23] |  | 0 | 0.01[0-0.024] | 0.12  [0-0.27] |
| *Chryseobacterium* | 0.04  [0.02-0.06] | 0.38  [0.03-0.73] | 0.03  [0.00-0.06] |  | 0.04  [0.02-0.07] | 0.003  [0-0.007] | 0.008  [0-0**.**01] |  | 0.02  [0.01-0.03] | 0.33  [0-0.69] | 0.05  [0-0.11] |
| *Micrococcus* | 0.08  [0.06-0.11] | 0.03  [0.005-0.021] | 0.21  [0.03-0.38] |  | 0.018  [0.009-0.02] | 0.003  [0.001-0.005] | 0.11  [0.06-0.15] |  | 0.03  [0.01-0.04] | 0.01  [0.004-0.02 | 0.10  [0.07-0.14] |
| *Bacteroides* | 0.004  [0-0.01] | 0 | 0.009  [0-0.03] |  | 0 | 0 | 0.21  [0-0.87] |  | 0 | 0 | 0.001  [0-0.004] |
| *Aeromonas* | 0.03  [0.004-0.068] | 0 | 0.002  [0-0.006] |  | 0.008  [0-0.02] | 0.006  [0-0.016] | 0.02  [0.003-0.048] |  | 0.01  [0-0.02] | 0 | 0.15  [0-0.42] |
