# Supplementary figures and images for: Dynamics of Bacterial Community Composition in the Malaria Mosquito's Epithelia
Source: Front Microbiol. 2016 Jan 5;6:1500. doi: 10.3389/fmicb.2015.01500 (PMC4700937; doi:10.3389/fmicb.2015.01500)

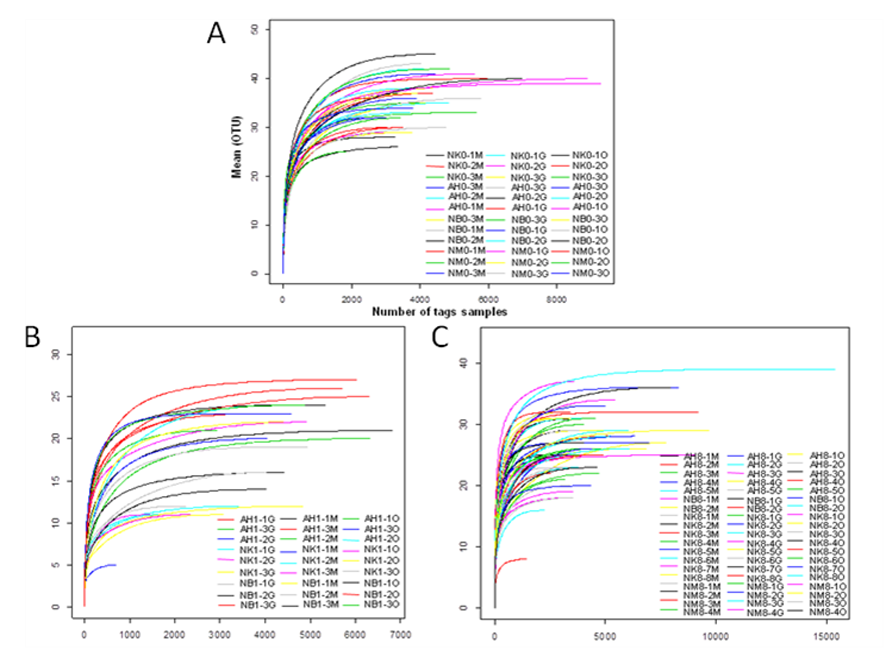

Supplement: Figure S1 — Rarefaction analyses for each mosquito sample. (A), emerging mosquitoes; (B), mosquitoes dissected at 24 h post blood feeding; (C), mosquitoes dissected 8 days post blood feeding. Saturation curves were generated by plotting the number of unique sequence tags as a function of the number of randomly sampled tags. Tags were clustered at k = 6 differences. NK, Nkolondom 10; NM, Nkolondom 11; NB, Nkolbisson; AH, Ahala; 0, samples from emerging mosquitoes; 1, samples from mosquitoes dissected at 24 h post blood feeding; 8, samples from mosquitoes dissected 8 days post blood feeding; M, midgut; O, ovaries; G, salivary glands. [file Image1.TIF]

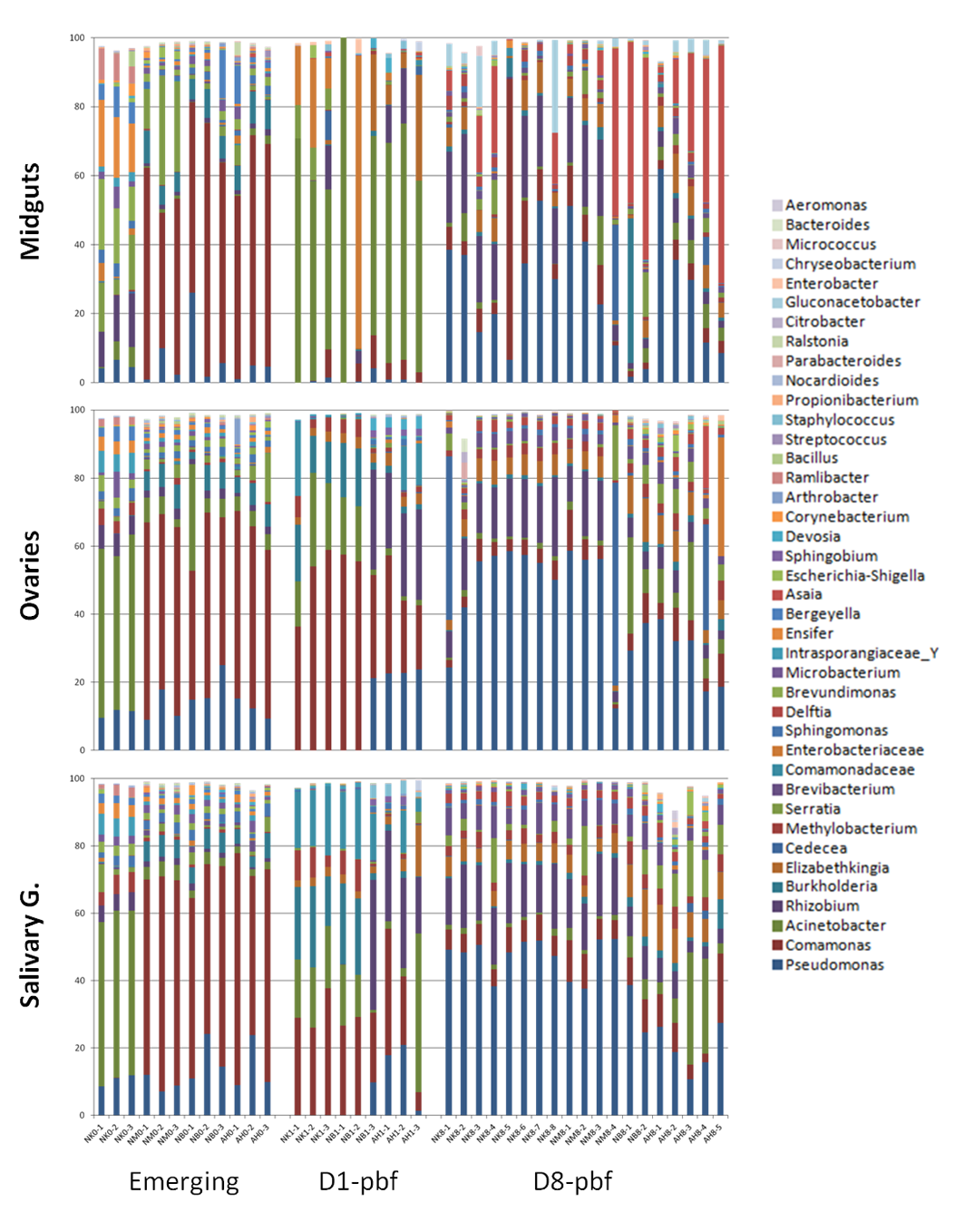

Supplement: Figure S2 — Taxonomic classification of bacterial reads retrieved from each individual mosquito in the different tissues at the distinct developmental stages. Emerging, emerging mosquitoes; D1-pbf, 24 h post blood feeding; D1-pbf, 8 days post blood feeding; NK, Nkolondom 10; NM, Nkolondom 11; NB, Nkolbisson; AH, Ahala. Relative abundances are given for the Genus level. [file Image2.TIF]
